# Sulfur-Doped Binary Layered Metal Oxides Incorporated on Pomegranate Peel-Derived Activated Carbon for Removal of Heavy Metal Ions

**DOI:** 10.3390/molecules27248841

**Published:** 2022-12-13

**Authors:** Binta Hadi Jume, Niloofar Valizadeh Dana, Marjan Rastin, Ehsan Parandi, Negisa Darajeh, Shahabaldin Rezania

**Affiliations:** 1 Department of Chemistry, College of Science, University of Hafr Al Batin, Al Jamiah District, P.O. Box 1803, Jeddah 39524, Saudi Arabia; 2 Department of Applied Chemistry, Faculty of Pharmaceutical Chemistry, Tehran Medical Sciences, Islamic Azad University, Tehran 1913674711, Iran; 3 Department of Metallurgy and Materials Engineering, Faculty of Engineering, University of Kashan, Kashan 8199696555, Iran; 4 Department of Food Science & Technology, Faculty of Agricultural Engineering and Technology, University of Tehran, Karaj 6719418314, Iran; 5 Department of Soil and Physical Sciences, Faculty of Agriculture and Life Sciences, Lincoln University, Lincoln, Christchurch 7647, New Zealand; 6 Department of Environment and Energy, Sejong University, Seoul 05006, Republic of Korea

**Keywords:** biomass, food waste, removal, heavy metal ions, nanocomposite, layered metal–sulfur

## Abstract

In this study, a novel biomass adsorbent based on activated carbon incorporated with sulfur-based binary metal oxides layered nanoparticles (SML-AC), including sulfur (S_2_), manganese (Mn), and tin (Sn) oxide synthesized via the solvothermal method. The newly synthesized SML-AC was studied using FTIR, FESEM, EDX, and BET to determine its functional groups, surface morphology, and elemental composition. Hence, the BET was performed with an appropriate specific surface area for raw AC (356 m^2^·g^−1^) and modified AC-SML (195 m^2^·g^−1^). To prepare water samples for ICP-OES analysis, the suggested nanocomposite was used as an efficient adsorbent to remove lead (Pb^2+^), cadmium (Cd^2+^), chromium (Cr^3+^), and vanadium (V^5+^) from oil-rich regions. As the chemical structure of metal ions is influenced by solution pH, this parameter was considered experimentally, and pH 4, dosage 50 mg, and time 120 min were found to be the best with high capacity for all adsorbates. At different experimental conditions, the AC-SML provided a satisfactory adsorption capacity of 37.03–90.09 mg·g^−1^ for Cd^2+^, Pb^2+^, Cr^3+,^ and V^5+^ ions. The adsorption experiment was explored, and the method was fitted with the Langmuir model (R^2^ = 0.99) as compared to the Freundlich model (R^2^ = 0.91). The kinetic models and free energy (<0.45 KJ·mol^−1^) parameters demonstrated that the adsorption rate is limited with pseudo-second order (R^2^ = 0.99) under the physical adsorption mechanism, respectively. Finally, the study demonstrated that the AC-SML nanocomposite is recyclable at least five times in the continuous adsorption–desorption of metal ions.

## 1. Introduction

Ions of heavy metals are frequently discovered in products generated by the oil industry. Vanadium (V^3+^), lead (Pb^2+^), chromium (Cr^3+^), and cadmium (Cd^2+^) are some examples of heavy metal ions that can be found [1]. Significant opportunities exist for these metal ions to enter groundwater, surface water, and drinking water because of a wide range of processes, including oil processing (extraction, shipping, and storage), mining, and other activities; as a result, the danger posed to the environment as well as to the health of humans is increased [2]. To prevent diseases and syndromes in humans, metabolic processes require trace amounts of heavy metals [3]. However, because heavy metals are used in a wide variety of industrial processes and are typically deposited in water, an excess amount of heavy metals can cause significant health issues in humans, such as degenerative processes in the muscles, body, and nervous system [3,4]. Therefore, monitoring and removing heavy metal ions from environmental water supplies is essential. The allowed levels for Cd^2+^, Cr^3+^, Pb^2+^, and V^5+^ in drinking water have been established at 5 μg L^−1^, 50 μg L^−1^, 10 μg L^−1^, and 100 mg L^−1^, respectively [2].

Several methods including bioremediation, electrocoagulation, reverse osmosis, oxidation, filtration, nanofibrous membrane, biochar, and adsorption [5,6,7,8] have been employed to remove heavy metal ions from water samples. Numerous benefits and drawbacks exist for each of these methods. For example, chemical precipitation creates hazardous waste, photocatalysis and reverse osmosis create secondary pollution, and ion exchange and filtering require advanced technology. Oxidation is also non-regenerative. Electrocoagulation and membrane technology are both intricate processes that result in unfavorable sludge. Even though bioremediation is a technology that is recognized to be safe, it is costly and cannot be applied in extreme conditions [5,6]. Due to their many advantages, researchers continue to be intrigued by adsorption-based approaches as highly efficient methods for treating water, especially for removing heavy metal ions. These advantages include the fact that they do not introduce secondary pollutants into the environment, are inexpensive, can be regenerated, require little complex equipment, and have high adsorption capacity [1,4,5].

Adsorbents for removing heavy metal ions have been made from various micro/nanoscale materials, including biomass, montmorillonite, carbon-based material, polymers, metal–organic framework, and metal oxides. Most proposed materials have drawbacks such as high price, lack of stability, insufficient sorption capacity, ineffective removal effectiveness, poor selectivity, and the generation of secondary pollutants [4,7]. Activated carbon (AC) is a highly porous adsorbent used in the adsorption process to control air and water pollution. This is because activated carbon possesses cationic and anionic nature [9], a high surface area, is cost-effective, has rapid biodegradability [10], is structurally reliable and thermally stable [2,6]. Coal-derived commercial activated carbon is commonly used to remove dissolved solids and ionic contaminants from potable water and industrial effluent, including heavy metal ions and synthetic colors. However, its high price, difficulty in regeneration, and disposal problems restrict its usefulness. Producing activated carbon from inexpensive agricultural products or materials (biochar) such as pomegranate peels and other food waste is one option to cut expenses [6]. There is a significant amount of potential for biochar AC as an adsorbent to be utilized in the process of modifying or reactivating the surface. The addition of metal oxide nanoparticles to AC can result in an improvement in both the efficiency and stability of the adsorption process [2].

The cations present between the layers of negatively charged metal-layered solids can be exchanged effectively. They are rigid and unaffected by temperature fluctuations. Additionally, compared to compounds with a single layer, they exhibit improved properties as a result of the synergistic interaction between the several phases [11]. The layers only have a weak connection to one another, but the ions that are intercalated between them help to keep them stable. The fact that these layered heterostructures have multiple functions makes them an extremely attractive topic of study. When distinct cations are intercalated, they can generate a wide variety of diverse characteristics and structures. They are less hazardous and less leachable due to features including biocompatibility, pH-dependent solvability, and the special intercalating property [2]. Metal-layered ion exchangers based on sulfides have remarkable adsorption and selectivity capabilities towards metal ions, and their removal kinetics are extremely fast. Highly selective and excellent ion exchange properties are displayed by inorganic ligands composed of sulfide metals because (1) the incorporated ions diffuse freely and can access the interior of the metal–ligand layers with relative ease and (2) the basic ligand forms strong interactions with the inserted metal ions [12]. Since soft Lewis basic sulfides have a high affinity for soft Lewis acidic metal ions, the ions are strongly adsorbed by the sulfides [2].

Herein, highly mesoporous activated carbon made from pomegranate peels was chemically precipitated with sulfur-based metal-layered manganese and tin ions to boost its adsorption capacity and efficiency. According to the literature reviews, the diffusion and nonisotopic exchanger processes are likely to be responsible for the ability of metal cation-doped adsorbent to selectively extract cations from complex matrix [2]. Utilizing FTIR, SEM, EDX, and BET to characterize the SML-AC nanocomposite, newly synthesized materials were employed as an adsorbent to remove Pb^2+^, Cd^2+^, Cr^3+^, and V^5+^ ions from oil-field water samples. Langmuir, Freundlich isotherm, and kinetic models were used to verify the experimental procedure and adsorption capacity.

## 2. Results and Discussion

### 2.1. Adsorbent Characterization

#### 2.1.1. FTIR Spectroscopy

**Figure 1:**
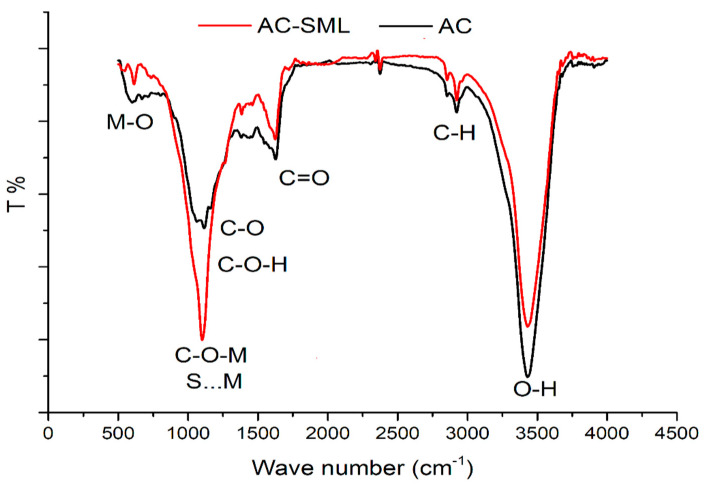
FTIR spectroscopy of pomegranate peel AC and AC-SML.

Surface functional groups of pristine AC and AC-SML were considered with FTIR, as shown in Figure 1. According to the AC spectrum, the band at 3400 cm^−1^ corresponds to the O-H groups anchored onto the carbon material. The IR bands at 2905 cm^−1^, 1712 cm^−1,^ and 1450 cm^−1^ and attributed to C-H, C=O, and C-C/C=C stretching of AC functionals and the skeleton of the carbon framework. After incorporating sulfur-layered metal oxide into AC, the IR spectrum of AC-SML showed a new sharp peak at 1020 cm^−1^ and small peaks at around 500 cm^−1^. It should be noted that the peaks that appeared in the wide range of 500–1050 cm^−1^ corresponded to the presence of metal–carbon (M-O-C) and sulfur–metal (M-S-M) linkage in the nanocomposite. This claim is confirmed by a previous study which demonstrated that the extensive bands 500–900 cm^−1^ are attributed to the metallic layered derived from the M-O stretching and O-M-O bonding [13,14]. Hence, the proposed characteristics of IR bands in both spectra of AC and AC-SML imply the successful incorporation of sulfur–metal layered into the activated carbon.

#### 2.1.2. SEM Microscopy

**Figure 2:**
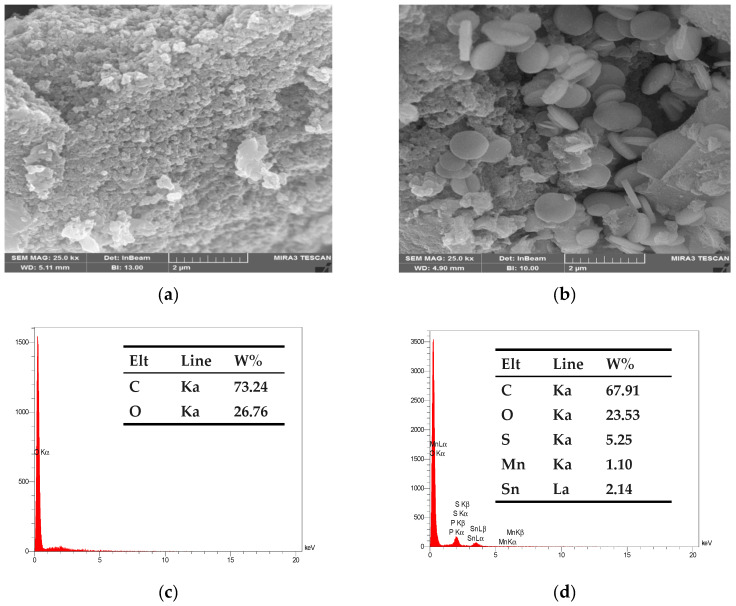
FESEM micrograph for raw nano AC (**a**) and modified AC-SML (**b**). EDX spectrum and elemental composition for raw AC (**c**) and AC-SML nanocomposite (**d**).

The newly synthesized pristine AC and AC-SML surface morphology were investigated using FESEM. Figure 2a illustrates the micrograph of AC. It is the external surface of the activated carbon which is quite irregular and full of cavities due to alkaline activation. After the immobilization of sulfur-layered metal oxide onto AC (Figure 2b), the micrograph indicates the formation of circular and bulk substances over AC. This trend confirms the formation of AC-SML nanocomposite.

#### 2.1.3. EDX Spectroscopy

To verify the presence of the preferred elements on plain AC and AC-SML nanocomposite, the EDX technique was utilized. Figure 2c,d represents the EDX signals and weight percentage of AC and AC-SML nanocomposite elements. The elemental analysis demonstrates the presence of two main elements over plain AC: carbon (73.24%) and oxygen (26.76%). Hence, after incorporating metal-S-oxide nanoparticles onto the AC surface, the expected elements were observed such as C, O, S, Mn, and Sn, with a weight percentage of 67.91%, 23.53%, 5.25%, 1.10%, and 2.14%, respectively. The EDX additionally performed the good dispersion of sulfur and layered metal (Sn-S-Mn) oxides on the activated carbon.

#### 2.1.4. BET Surface Area

**Figure 3:**
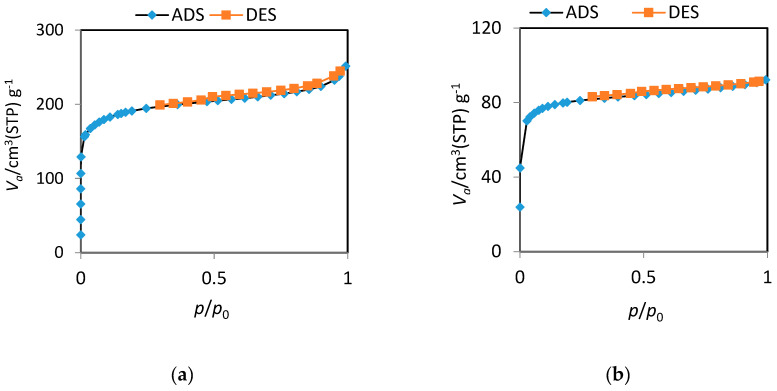
BET-N_2_ adsorption-desorption process for plain AC (**a**) and AC-SML nanocomposite (**b**).

Specific surface area is an imperative factor in adsorption chemistry since it directly reflects the sorption capacity. BET technique based on the N_2_ adsorption–desorption process utilized to record the surface area of plain AC and AC-SML nanocomposite. The N_2_ adsorption–desorption curve is shown in Figure 3 for AC (a) and AC-SML nanocomposite (b). The specific surface area values were obtained at 356 m^2^·g^−1^ and 195 m^2^·g^−1^, respectively. The specific surface area is decreased after incorporating sulfur-based metal oxide into the AC. The high specific surface area value for plain AC is probably due to the porous structure, which also provided a higher pore diameter (3.38 nm) than the modified nanocomposite (2.92 nm). The pore volume and diameter indicate that the prepared materials are mesoporous, which is appropriate for adsorption.

### 2.2. Adsorption Parameters

#### 2.2.1. Types of Materials

**Figure 4:**
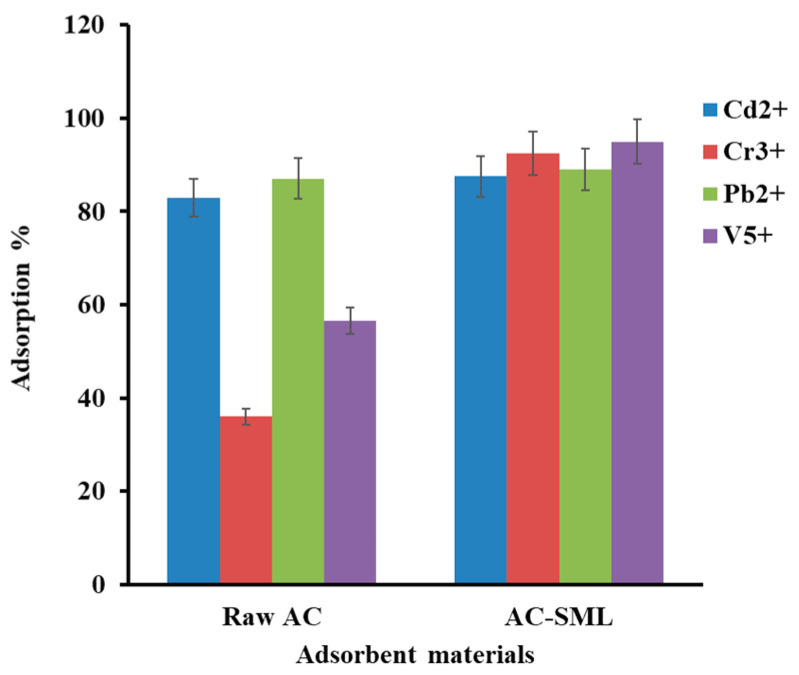
Influence types of materials on adoption efficiency.

The influence of the types of adsorbent material (raw Ac and AC-SML) on adsorbent efficiency was studied in similar conditions (dosage of 100 mg, pH 4, and time 60 min). According to Figure 4, the raw AC shows high efficiency for Cd^2+^ and Pb^2+^ ions and low efficiency for Cr^3+^ and V^5+^. This probably is due to the occupation of AC active sites with Cd^2+^ and Pb^2+^ ions, since it is rich in oxygenated functional groups. Moreover, after modification of AC with doping SML (Sn-S-Mn) nanoparticles, an increment in the adsorption percentage was observed for selected metal ions (Cd^2+^, Cr^3+^, Pb^2+^, and V^5+^). This phenomenon can be associated with the enhancement of the electrostatic interaction between sulfur (S^2−^) and a selected metal cation. Hence, the trend (Figure 4) reflects the synergetic effect of AC-SML nanocomposite for the uptake of Cd^2+^, Cr^3+^, Pb^2+^, and V^5+^ cations.

#### 2.2.2. Effect of Solution pH

**Figure 5:**
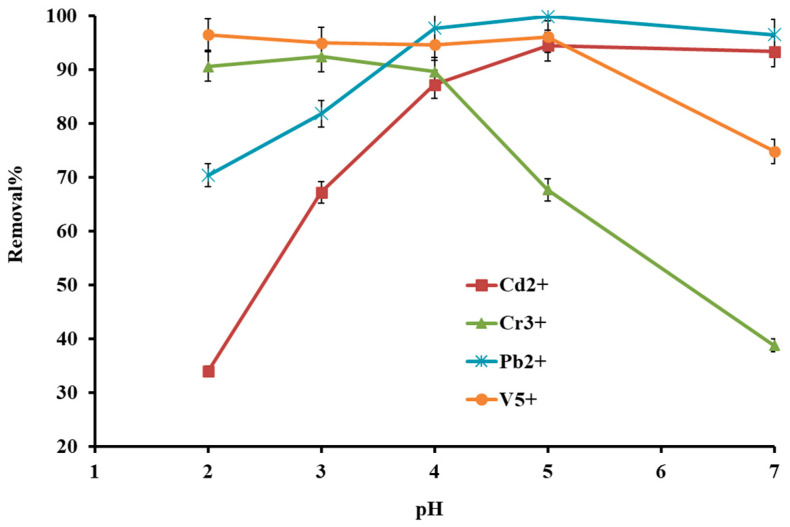
Effect of solution pH on metal ion removal efficiency.

The solution pH was investigated from 2 to 7 since metal ions are highly influenced by solution pH. In the survey literature, the selected metal ions are existing in different states, including cadmium: 2–8.5 (Cd^2+^), 8.6–10.5 (Cd(OH)^−^), 10.6–12 (Cd(OH)_2_)_aq_ [15]; chromium: 1–3 (Cr^3+^), 3.1–6 (Cr(OH^2+^)), 6.1–8 (Cr(OH^+^)_2_), 8–2 (Cr(OH)_3_) [16]; lead: 1–6.1 (Pb^2+^), 6.2–8.5 (Pb(OH)^−^), 8.6–11 (Pb(OH)_2_ aq) [17], and vanadium: pH 2–3 (VO_2_), 3.1–4 (VO(OH_3_)^0^), 4.1–8.5 (VO_2_(OH)_2_^−^), 8.6–10 (VO_3_(OH)^2−^). Removal efficiency is experimentally investigated for selected heavy metal ions and shown in Figure 5. As can be seen, Cd^2+^ and Pb^2+^ provided low efficiency at low pH, which can be defined through the undesired protonation of the adsorbent surface followed by electrostatic repulsion. It is also noteworthy that the proton ions (H^+^) trade competition with metal cations to adsorb over nanocomposite. Due to electrostatic interaction and coordination, the favorable removal efficiency was obtained for Cd^2+^ and Pb^2+^ at pH 4 to 6. A slight decrease in efficiency at pH 6–7 is due to the formation of metal hydroxide (Pb(OH)^−^). Chromium and vanadium showed different trends due to their neutralization in the presence of a hydrogen wealthy medium. The other reason that may additionally interact in such pH-dependent behavior is the specific constructions and geometries of the metal cations with pH, which increased the adsorption efficiency. However, pH 4 was the best experimental condition for all metal cations.

#### 2.2.3. Effect of Adsorbent Dosage

**Figure 6:**
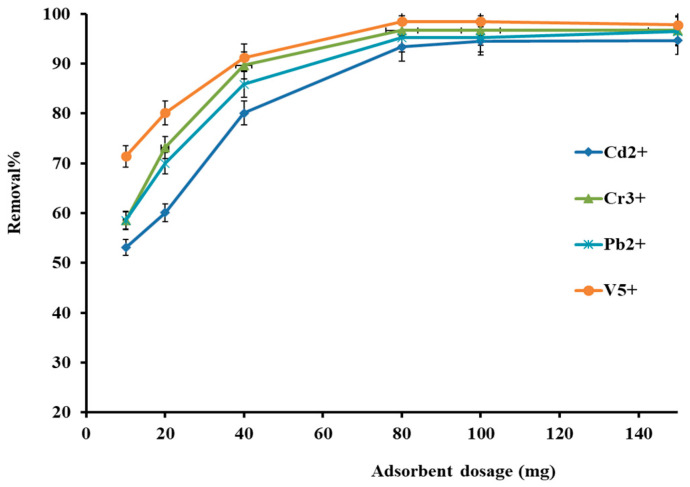
Effect of AC-SML dosage on metal ion removal efficiency.

The amount of adsorbent is vital for the quantitative removal of adsorbate in the adsorption process. This is described via the adsorbent dosage in the range of 10–150 mg, as shown in Figure 6. The removal profile reflected that the adsorption efficiency is increasing for all selected metal cations. However, the quantitative adsorption efficiency increased from 52% to 95% when sorbent material varied from 10 to 80 mg. After that, the efficiency slightly changed up to 150 mg. Hence, the efficiency expansion can be attributed to more adsorption sites on the adsorbent since the solid phase surface area increases exponentially with the regular degree of adsorbent dosage. Thus, 80 mg was selected for all metal cations with maximum adsorption efficiency.

#### 2.2.4. Effect of Time

**Figure 7:**
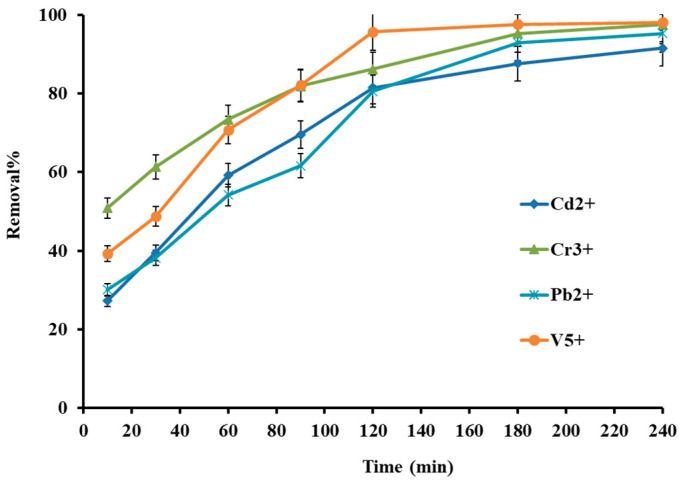
Effect of contact time on metal ion removal efficiency.

Adsorption’s contact time is another crucial parameter for the effective uptake of metal ions from an aqueous solution. The impact of contact time for the adsorption of the selected heavy metal cation was investigated in the exclusive times in the range of 5–240 min. Figure 7 indicates that the removal increased gradually from 25% to 80% for all metal cations by increasing the time up to 120 min, except vanadium with 96%. With additional increase in the contact time up to 240 min, adsorption efficiency no longer extends significantly for vanadium, but the adsorption efficiency of lead, cadmium, and chromium ions increases to >92%. The first stage (5–120 min) is performing the availability of the active site to uptake metal cations, and after that, the adsorption sites are saturated, and the system reaches equilibrium [18]. Finally, the 120 min was selected for the further procedure due to the high percent removal.

### 2.3. Adsorption Kinetics

The adsorption time limitation is performed with kinetic models of pseudo-first-order and pseudo-second-order models. The linear varieties of the proposed kinetic models can be estimated with Equations (1) and (2), respectively.

(1) $$\mathrm{Ln}\left(Q_{e}-Q_{t}\right)={\mathrm{LnQ}}_{e}-k_{1}t,$$

(2) tQt=1k2Qe2+tQe

The parameters are the following: Q_e_ (mg/g) is equilibrium adsorption capacity, Q_t_ (mg/g) adsorption capability at any time t. The time constates of k_1_ (1/min) and k_2_ (g/mg/min) corresponds to the first-order and second-order models, respectively.

**Figure 8:**
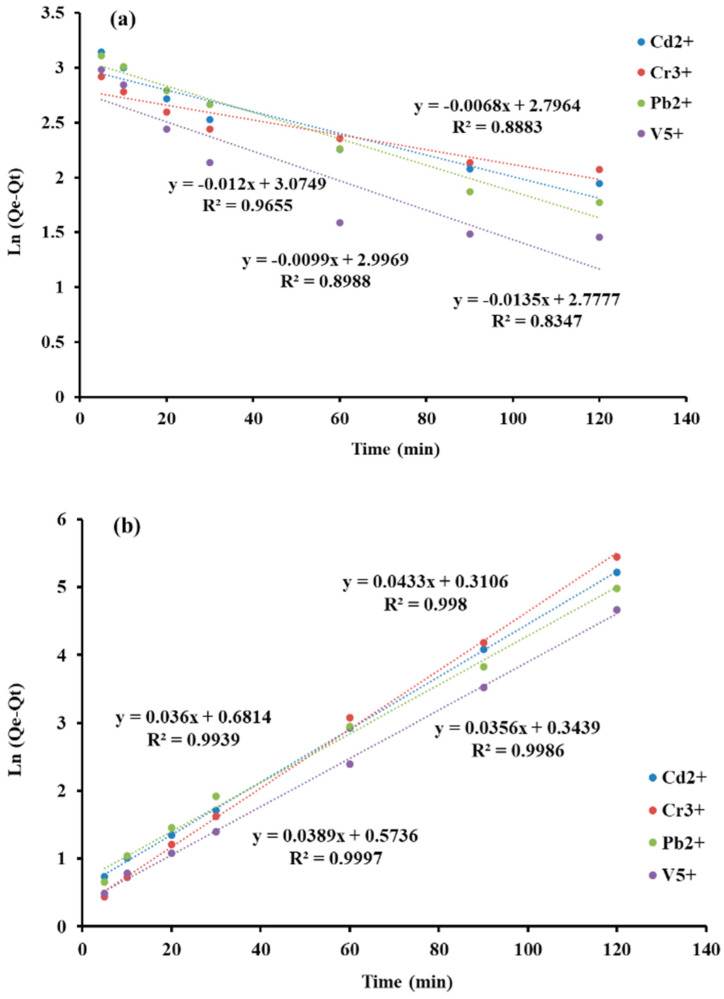
Kinetic models of pseudo-first order (**a**) and pseudo-second order (**b**).

Figure 8a,b reflects the linear plots of kinetic models, which are plotted Ln(Q_e_−Q_t_) versus time and t/Q_t_ versus time, and the values of parameters are listed in Table 1. Based on the determination coefficient (R^2^), the contact time was fitted with pseudo-second order (>0.99) as compared to pseudo-first order (0.88–0.96). In addition, Q_e_ values (theory) of second-order models were in good agreement with experimental adsorption capacity. Therefore, the kinetic rate is limited with pseudo-second order, in which the adsorption process has been controlled with electron sharing and electrostatic interactions.

### 2.4. Adsorption Equilibrium and Isotherm Models

**Figure 9:**
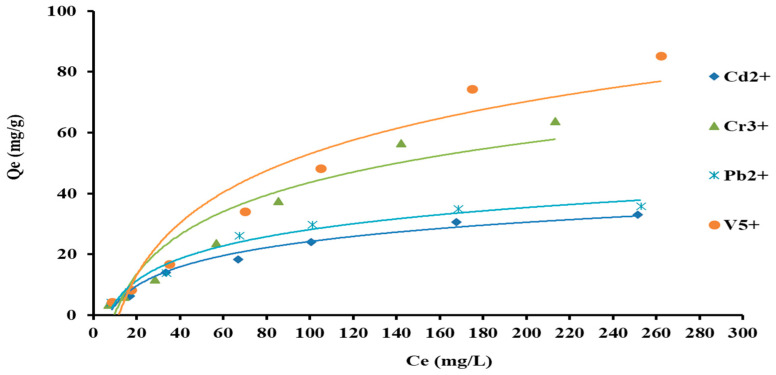
Equilibrium adsorption capacity versus the initial concentration.

The impact of the initial concentration of selected heavy metal ions is experimented on to consider the equilibrium capability of AC-SML nanocomposite. For this, 10 mg of AC-SML was applied to adsorb the Cd^2+^, Cr^3+^, Pb^2+,^ and V^5+^ with a concentration range of 5–300 mg·L^−1^ in 120 min contact time. The equilibrium isotherm (Q_e_ vs. C_e_) was recorded and plotted as shown in Figure 9. The isotherm graph shows the expected Qe increases from 3 mg·g^−1^ to 85 mg·g^−1^ via increasing the concentration of selected heavy metal ions until it reaches equilibrium. The isothermal evaluation revealed that the adsorption process follows the IUPAC regular pattern (Type II) [19]. This pattern described the adsorption process following a monolayer pattern.

Hence, isotherm models were utilized to explore the adsorption pattern and mechanism. Therefore, the well-known isotherm models, namely Langmuir, Freundlich, Dubinin–Radushkevich (DR), and free energy were used to describe the adsorption pattern and adsorption mechanism. Langmuir is attributing the monolayer adsorption onto the homogenous surface. Freundlich reflects the multilayer adsorption onto the heterogeneous surface. DR confirms the multilayer adsorption over either homogenous or heterogeneous surfaces. Free energy suggests the adsorption of machoism, physical or chemical. Hence, the proposed models’ linear mechanism formed by the following Equations (3)–(5):

(3) CeQe=CeQm+1KLQm

(4) lnQe=lnKF+(1n)lnCe

(5) $$\ln Q_{e}=\ln Q_{S}-K_{\mathrm{ad}}\left(\varepsilon^{2}\right)$$

(6) $$\varepsilon =\mathrm{RT}\;\ln\;\left(1+1/C_{e}\right)$$

(7) $$E={\left(2K_{\mathrm{ad}}\right)}^{-1/2}$$

In the equations, Q_m_ (mg·g^−1^) is maximum adsorption capacity, K_L_ (L·mg^−1^), K_ad,_ and K_F_ [(mg/g)(L/mg)^1/n^] correspond to Langmuir, DR, and Freundlich constants, respectively. C_e_ (mg·L^−1^) equilibrium concentration of heavy metal ions and 1⁄n reflect the favorability of the adsorption process. Q_s_ (mg·g^−1^) is the DR theoretical sorption capacity. Finally, E is sorption energy (Equation (7)), directly displaying the adsorption mechanism. The E values < 40 kJ/mol are the physisorption mechanism, and E > 80 kJ/mol is the chemisorption mechanism [20,21].

**Figure 10:**
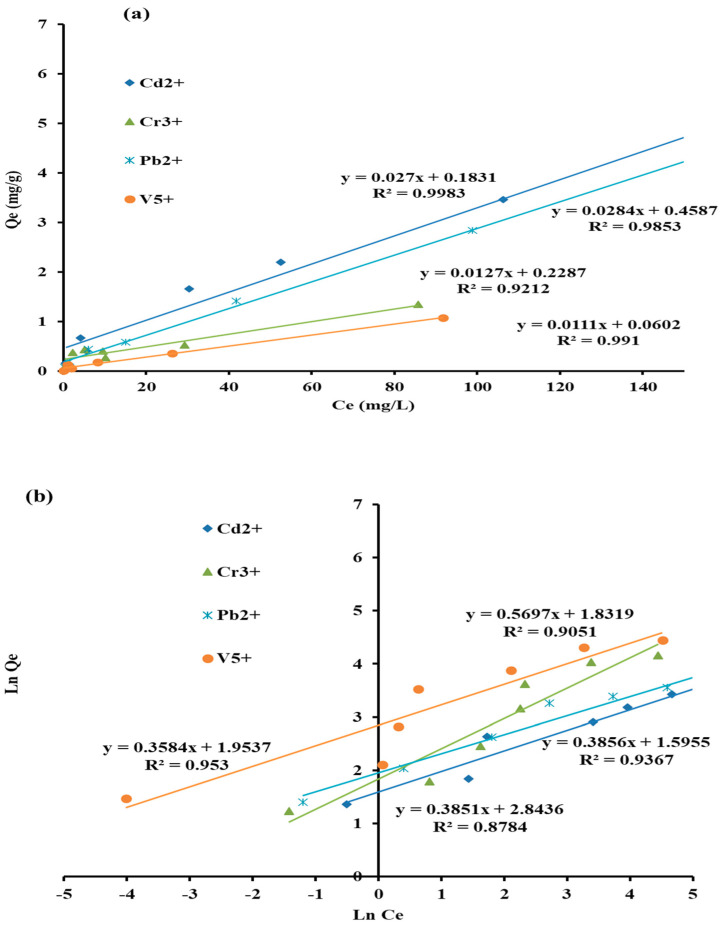
Adsorption isotherm models of Langmuir (**a**) and Freundlich (**b**).

The corresponding linear models of every isotherm have been plotted (Figure 10a,b), and the parameters of every model were calculated in Table 2. Based on the values of R^2^, it used to be assumed that both Langmuir (R^2^ > 0.92) and Freundlich (R^2^ > 0.90) models observe to describe the adsorption of selected heavy metal ions onto the AC-SML adsorbent nanocomposite. Hence, adsorption can be either monolayer or multilayer, with a maximum adsorption capacity of 37.03 mg·g^−1^ to 90.09 mg·g^−1^ for all selected metal ions. Moreover, the appropriate values of R^2^ of the DR model confirm the favourability of the multilayer pattern. The values of energy approved the physisorption mechanism for both monolayer and multilayer adsorption patterns for selected heavy metal ions over AC-SML nanocomposite.

### 2.5. Mechanism Study

**Figure 11:**
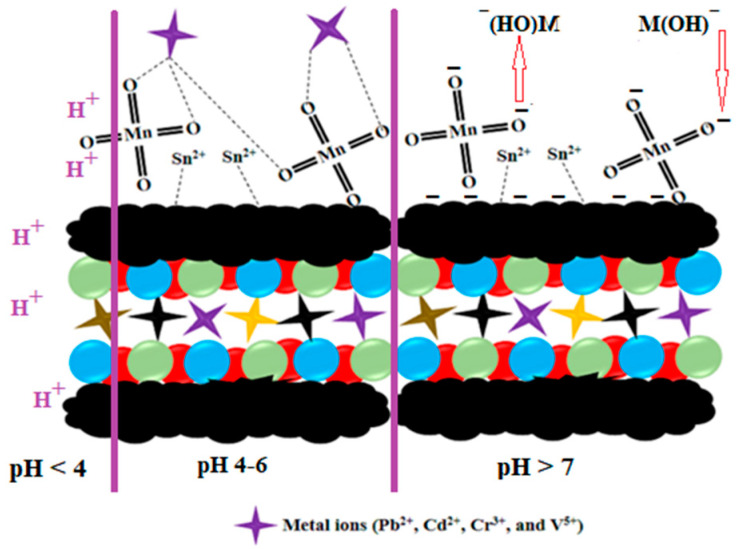
Schematic of the proposed mechanism for heavy metal ion removal by AC-SML.

The proposed adsorption mechanism between the selected heavy metal ions and AC-SML is investigated, as graphically shown in Figure 11. Hence, based on the pH study, high efficiency was obtained in the pH ranges of 4–6. Therefore, the possible electrostatic interactions and coordination between Cd^2+^, Cr^3+^, Pb^2+,^ and V^5+^ and functional groups (sulfur-metal) of the AC could be the reason for the high removal efficiency at pH 4–6. In addition, the lower removal efficiency at low pH (<4) may be because of the AC-SML’s protonation of active sites as well as the competition of H^+^ for adsorption, while at high pH > 7, the selected metal cations are present as hydroxide form (M(OH_x_)^−^), which is the prominent repulsion force interactive between the adsorbates and negative surface charge of adsorbent (AC-SML^-^).

### 2.6. Regeneration

To study the regeneration, 80 mg of the AC-SML was positioned into a test tube, including selected Cd^2+^, Cr^3+^, Pb^2+,^ and V^5+^. After each adsorption process, the adsorbates were desorbed using 5 mL (HCl, 0.1 M) after being shaken for 30 min. Then, launched heavy metal ions were measured with ICP-OES. Then, the AC-SML was washed with excess distilled water and reused for a further experimental process for repeated five adsorption–desorption cycles. The adsorption efficiency was obtained 85%, 81%, 87%, 80% for Cd^2+^, Cr^3+^, Pb^2+^ and V^5+^, respectively. This procedure indicated that the removal effectivity of selected heavy metal ions does not reduce notably for up to five cycles. Thus, it can be claimed that the AC-SML nanocomposite can be regenerated for five adsorption–desorption cycles.

### 2.7. Comparison

Table 3 indicates that the freshly synthesized AC-SML nanocomposite could be noteworthy for removing Cd^2+^, Cr^3+^, Pb^2+,^ and V^5+^ ions. Compared to other adsorbents, the AC-SML nanocomposite showed high adsorption capacity at pH 4. The adsorption process duration was relatively fast. The AC-SML’s improved adsorption capacity was due to the doping of the sulfur metal layer oxides on the AC, enhancing interactions with heavy metal ions and resulting in significant removal.

## 3. Material and Methods

### 3.1. Material

Tin (II) chloride (SnCl_2_), manganese (II) chloride tetrahydrate (MnCl_2_·4H_2_O), chromium (III) nitrate nonahydrate (Cr(NO)_3_·9H_2_O), cadmium chloride hemipentahydrate (CdCl_2_·2.5H_2_O), lead nitrate (Pb(NO_3_)_2_), ammonium metavanadate (NH_4_VO_3_), hydrochloric acid (HCl, 37%), sodium hydroxide (NaOH), and sodium chloride (NaCl) were purchased from Merck Chemicals.

### 3.2. Adsorbent Synthesis

#### 3.2.1. Pretreatment and Synthesis AC

The nanosized pomegranate peel-activated carbon was prepared according to the previous study [9]. Briefly, the raw pomegranate peel (PG) was washed and dried in the laboratory under ambient temperature and dark place. The dried and clean pomegranate peel was ground into a fine powder and sieved properly (<150 μm). Then, lignans were extracted by combining 20 g of pomegranate peel powder with 6 g of NaOH in 200 mL of distilled water and stirring for 24 h. After being diluted and filtered through filter paper, the mixture was rinsed with excess distilled water and finally dried in an oven at a temperature of 85 °C. Next, the uniformly sized powder was carbonized in a furnace at 400 °C for 3 h (under an N_2_ atmosphere) to produce biochar. Using a mass ratio of 5:1 (AC/NaOH), 120 mL of distilled water, and vigorously stirring at 120 °C for 2 h, we thoroughly combined the produced carbon with the NaOH solution. Following filtration of the solution, the isolated product was dried in an oven at 120 °C for 10 h. It was transferred to the furnace to finish the activation process and heated to 800 °C for 2 h. After being neutralized in 0.01 M HCl and excess distilled water, AC was dried in an oven at 90 °C for 24 h [2].

#### 3.2.2. Synthesis of SML-AC

**Figure 12:**
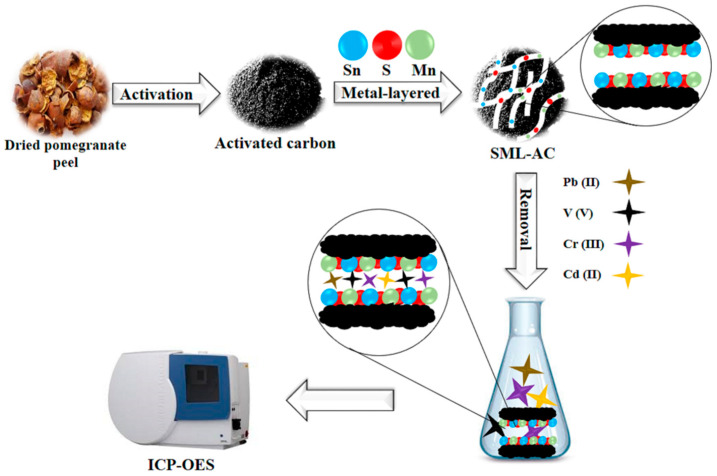
A schematic for SML-AC synthesis and removal procedure.

Subsequently, 1 g S_2_, 0.1 g SnCl_2_, and 0.1 g MnCl_2_ were added to a solution containing 2 g of the dried AC in 150 mL of distilled water, and the combination was agitated at 45 °C for 3 h (pH 9). Afterward, the solid was separated from the mixture and heated in the furnace for 2 h at 650 °C. The product (SML-AC) was then rinsed with extra distilled water and dried in an oven at 85 °C for 18 h [23,24,25] (Figure 12).

### 3.3. Adsorbent Characterization

A Bruker Equinox 55 spectrometer was used to conduct Fourier-transform infrared spectroscopy (*FTIR*) in the 400–4000 cm^−1^ wavenumber region to identify the functional groups of the newly synthesized material. The surface morphology and elemental structure of the AC and SML-AC were examined using a field emission scanning electron microscopy (FESEM) TESCAN MIRA3 equipped Energy Dispersive X-ray Analysis (EDX). Brunauer–Emmett–Teller (BET) determined the surface area and pore size.

### 3.4. Removal Procedure

First, 30 mL of the sample solution that contained 30 mg·L^−1^ of the target analytes mixed with 40 mg of the SML-AC adsorbent. After 30 min of orbital shaking on a shaker, the mixture was then centrifuged to separate the SML-AC from the mixture (4000 rpm, 6 min). Lastly, a syringe filter made of cellulose with a mesh size of 0.2 was used to filter 5 mL of the supernatant, which included the metal ions in residual concentration. This sample was immediately subjected to an inductively coupled plasma optical emission spectroscopy (ICP-OES) analysis. In the adsorption trials, multiple critical parameters such as pH (ranging from 2 to 7), SML-AC quantity (ranging from 10 to 150 mg), and contact duration (ranging from 5 to 240 min) were explored and improved to produce acceptable results from the adsorbent. Required parameters were calculated below [1,26].

(8) $$\mathrm{Removal}\;\mathrm{efficiency}\;\left(R\%\right)=\left(\frac{C_{1}-C_{e}}{C_{1}}\right)\times 100$$

(9) $$\mathrm{Adsorption}\;\mathrm{capacity}\;\left(q_{e}\right)=\left(\frac{V}{m}\right)\times \left(C_{1}-C_{e}\right)$$

*C*_1_ and *C_e_* are initial and residual concentrations of ions (mg·L^−1^), respectively. *V* is the initial sample volume (mL), *q_e_* is the equilibrium adsorption capacity (mg·g^−1^), and *W* represents the adsorbent dosage (mg).

## 4. Conclusions

This study developed an efficient nano-sized adsorbent for removing heavy metal ions from aqueous solutions using a metal–sulfur-layered oxide immobilized on pomegranate peel-derived activated carbon. The nanocomposite was prepared and used for removing selected heavy metal ions (Cd^2+^, Cr^3+^, Pb^2+^, and V^5+^) from the water sample at pH 4. The equilibrium isotherm models were used to explain the adsorption capacity, which obtained 37.03, 78.74, 35.21, and 90.09 mg·g^−1^ for Cd^2+^, Cr^3+^, Pb^2+^, and V^5+^, respectively. The Langmuir and Freundlich model and free Energy findings were carried out in the selected heavy metal ions adsorption process following monolayer and multilayer sorption patterns underneath the physical adsorption process. The kinetic rate was limited with pseudo-second order followed by electron sharing and electrostatic interactions. Hence, with a high removal efficiency of >90% for all selected heavy metal ions, the AC-SML nanocomposite is an efficient adsorbent for removing Cd^2+^, Cr^3+^, Pb^2+,^ and V^5+^ from aqueous media.

## Figures and Tables

**Table 1:** Kinetic models and parameters for selected heavy metal ions.

Kinetic Models	Parameters	Cd^2+^	Cr^3+^	Pb^2+^	V^5+^
**pseudo-first order**	*R* ^2^	0.898	0.888	0.965	0.834
	*k*_1_ (1/min)	0.009	0.006	0.0121	0.0135
	*q_e_* (mg·g^–1^)	19.16	16.74	22.07	16.31
**pseudo-second order**	*R* ^2^	0.999	0.998	0.993	0.998
	*k*_2_ (g/mg/min)	0.0012	0.0018	0.0012	0.0015
	*q_e_* (mg·g^–1^)	25.71	23.25	27.77	28.08

**Table 2:** Langmuir, Freundlich, DR models and free Energy for adsorption of selected heavy metal ions over AC-SML nanocomposite.

Isotherms	Parameters	Cd^2+^	Cr^3+^	Pb^2+^	V^5+^
**Langmuir**	*Q_m_* (mg g^−1^)	37.03	78.74	35.21	90.09
	*k_L_* (L mg^−1^)	0.144	0.055	0.061	0.184
	*R* ^2^	0.998	0.921	0.985	0.991
**Freundlich**	*K_F_* [(mg g^−1^) (L mg^−1^)^1/n^]	4.42	5.51	6.17	14.11
	*n*	2.59	1.75	2.79	2.59
	*R* ^2^	0.936	0.905	0.953	0.878
**Dubinin–Radushkevich**	*Q_s_* (mg g^−1^)	18.61	24.98	22.39	38.29
	*K_ad_* (mol^2/^kJ^2^)	2.908	2.161	0.736	0.592
	*R^2^*	0.864	0.848	0.886	0.903
**Energy**	*E* (kJ/mol)	0.41	0.48	0.82	0.91

**Table 3:** Comparison of AC-SML with other published adsorbents for heavy metal sorption.

Adsorbent	Heavy Metals	pH	Time (min)	Qe (mg/g)	Ref.
AC-SML	Pb^2+^, Cd^2+^, Cr^3+^, V^5+^	4	120	37–90	This study
AC-metal phosphate layered	Pb^2+^, Cd^2+^, Co^2+^, V^5+^, Ni^2+^	6	140	20–140	[2]
AC-silica	Pb^2+^, Cd^2+^, Ni^2+^	7	200	61–400	[22]
Magnetic sporopollenin-polyaniline	Pb^2+^	6	90	163.93	[5]
Magnetic graphene oxide-TiLa	Pb^2+^	5	120	112	[4]
Alginate@MgS	Pb^2+^	4	140	84.74	[1]

## Data Availability

Not applicable.

## Sample Availability

Samples of the adsorbent are available from the authors.

## References

[1] Bidhendi M.E., Parandi E., Mahmoudi-Meymand M., Sereshti H., Nodeh H.R., Joo S.-W., Vasseghian Y., Khatir N.M., Rezania S. (2022). Removal of lead ions from wastewater using magnesium sulfide nanoparticles caged alginate microbeads. Environ. Res..

[2] Esmaeili Bidhendi M., Abedynia S., Mirzaei S.S., Gabris M.A., Rashidi Nodeh H., Sereshti H. (2020). Efficient removal of heavy metal ions from the water of oil-rich regions using layered metal-phosphate incorporated activated carbon nanocomposite. Water Environ. J..

[3] Nafi A.W., Taseidifar M. (2022). Removal of hazardous ions from aqueous solutions: Current methods, with a focus on green ion flotation. J. Environ. Manag..

[4] Mosleh N., Ahranjani P.J., Parandi E., Nodeh H.R., Nawrot N., Rezania S., Sathishkumar P. (2022). Titanium lanthanum three oxides decorated magnetic graphene oxide for adsorption of lead ions from aqueous media. Environ. Res..

[5] Mosleh N., Najmi M., Parandi E., Nodeh H.R., Vasseghian Y., Rezania S. (2022). Magnetic sporopollenin supported polyaniline developed for removal of lead ions from wastewater: Kinetic, isotherm and thermodynamic studies. Chemosphere.

[6] Vonnie J.M., Li C.S., Erna K.H., Yin K.W., Felicia W.X.L., Aqilah M.N.N., Rovina K. (2022). Development of Eggshell-Based Orange Peel Activated Carbon Film for Synergetic Adsorption of Cadmium (II) Ion. Nanomaterials.

[7] Lilhare S., Mathew S.B., Singh A.K., Carabineiro S.A. (2022). Aloe Vera Functionalized Magnetic Nanoparticles Entrapped Ca Alginate Beads as Novel Adsorbents for Cu (II) Removal from Aqueous Solutions. Nanomaterials.

[8] Parandi E., Pero M., Kiani H. (2022). Phase change and crystallization behavior of water in biological systems and innovative freezing processes and methods for evaluating crystallization. Discov. Food.

[9] Esmaeili Bidhendi M., Poursorkh Z., Sereshti H., Rashidi Nodeh H., Rezania S., Afzal Kamboh M. (2020). Nano-size biomass derived from pomegranate peel for enhanced removal of cefixime antibiotic from aqueous media: Kinetic, equilibrium and thermodynamic study. Int. J. Environ. Res. Public Health.

[10] Dhahri R., Yılmaz M., Mechi L., Alsukaibi A.K.D., Alimi F., ben Salem R., Moussaoui Y. (2022). Optimization of the Preparation of Activated Carbon from Prickly Pear Seed Cake for the Removal of Lead and Cadmium Ions from Aqueous Solution. Sustainability.

[11] Venugopal B., Rajamathi M.J.J.o.c. (2011). Layer-by-layer composite of anionic and cationic clays by metathesis. J. Colloid Interface Sci..

[12] Manos M.J., Kanatzidis M.G. (2016). Metal sulfide ion exchangers: Superior sorbents for the capture of toxic and nuclear waste-related metal ions. Chem. Sci..

[13] Sarma D., Malliakas C.D., Subrahmanyam K., Islam S.M., Kanatzidis M.G. (2016). K_2x_Sn _4−x_S_8−x_ (x = 0.65–1): A new metal sulfide for rapid and selective removal of Cs^+^, Sr^2+^ and UO_2_^2+^ ions. Chem. Sci..

[14] Yadollahi M., Namazi H. (2013). Synthesis and characterization of carboxymethyl cellulose/layered double hydroxide nanocomposites. J. Nanopart. Res..

[15] Bian Y., Bian Z., Zhang J., Ding A., Liu S., Zheng L., Wang H. (2015). Adsorption of cadmium ions from aqueous solutions by activated carbon with oxygen-containing functional groups. Chin. J. Chem. Eng..

[16] Santos V.C.G.D., Salvado A.d.P.A., Dragunski D.C., Peraro D.N.C., Tarley C.R.T., Caetano J. (2012). Highly improved chromium (III) uptake capacity in modified sugarcane bagasse using different chemical treatments. Química Nova.

[17] Duan S., Tang R., Xue Z., Zhang X., Zhao Y., Zhang W., Zhang J., Wang B., Zeng S., Sun D.J.C. (2015). Effective removal of Pb (II) using magnetic Co_0.6_Fe_2.4_O_4_ micro-particles as the adsorbent: Synthesis and study on the kinetic and thermodynamic behaviors for its adsorption. Colloids Surf. A Physicochem. Eng. Asp..

[18] Naushad M. (2014). Surfactant assisted nano-composite cation exchanger: Development, characterization and applications for the removal of toxic Pb^2+^ from aqueous medium. Chem. Eng. J..

[19] Sing K.S. (1985). Reporting physisorption data for gas/solid systems with special reference to the determination of surface area and porosity (Recommendations 1984). Pure Appl. Chem..

[20] Saleh T.A. (2015). Isotherm, kinetic, and thermodynamic studies on Hg (II) adsorption from aqueous solution by silica-multiwall carbon nanotubes. Environ. Sci. Pollut. Res..

[21] Mona S., Kaushik A. (2015). Chromium and cobalt sequestration using exopolysaccharides produced by freshwater cyanobacterium Nostoc linckia. Ecol. Eng..

[22] Karnib M., Kabbani A., Holail H., Olama Z. (2014). Heavy metals removal using activated carbon, silica and silica activated carbon composite. Energy Procedia.

[23] Shirani M., Aslani A., Sepahi S., Parandi E., Motamedi A., Jahanmard E., Nodeh H.R., Akbari-Adergani B. (2022). An efficient 3D adsorbent foam based on graphene oxide/AgO nanoparticles for rapid vortex-assisted floating solid phase extraction of bisphenol A in canned food products. Anal. Methods.

[24] Shirani M., Parandi E., Nodeh H.R., Akbari-Adergani B., Shahdadi F. (2022). Development of a rapid efficient solid-phase microextraction: An overhead rotating flat surface sorbent based 3-D graphene oxide/lanthanum nanoparticles@ Ni foam for separation and determination of sulfonamides in animal-based food products. Food Chem..

[25] Aghel B., Gouran A., Parandi E., Jumeh B.H., Nodeh H.R. (2022). Production of biodiesel from high acidity waste cooking oil using nano GO@ MgO catalyst in a microreactor. Renew. Energy.

[26] Parandi E., Safaripour M., Abdellattif M.H., Saidi M., Bozorgian A., Nodeh H.R., Rezania S. (2022). Biodiesel production from waste cooking oil using a novel biocatalyst of lipase enzyme immobilized magnetic nanocomposite. Fuel.

